# Quantifying the Effects of Topology and Weight for Link Prediction in Weighted Complex Networks

**DOI:** 10.3390/e20050363

**Published:** 2018-05-13

**Authors:** Bo Liu, Shuang Xu, Ting Li, Jing Xiao, Xiao-Ke Xu

**Affiliations:** 1 College of Information and Communication Engineering, Dalian Minzu University, Dalian 116600, China; 2 Guizhou Provincial Key Laboratory of Public Big Data, Guizhou University, Guiyang 550025, China

**Keywords:** weighted networks, link prediction, null models

## Abstract

In weighted networks, both link weight and topological structure are significant characteristics for link prediction. In this study, a general framework combining null models is proposed to quantify the impact of the topology, weight correlation and statistics on link prediction in weighted networks. Three null models for topology and weight distribution of weighted networks are presented. All the links of the original network can be divided into strong and weak ties. We can use null models to verify the strong effect of weak or strong ties. For two important statistics, we construct two null models to measure their impacts on link prediction. In our experiments, the proposed method is applied to seven empirical networks, which demonstrates that this model is universal and the impact of the topology and weight distribution of these networks in link prediction can be quantified by it. We find that in the *USAir*, the *Celegans*, the *Gemo*, the *Lesmis* and the *CatCortex*, the strong ties are easier to predict, but there are a few networks whose weak edges can be predicted more easily, such as the *Netscience* and the *CScientists*. It is also found that the weak ties contribute more to link prediction in the *USAir*, the *NetScience* and the *CScientists*, that is, the strong effect of weak ties exists in these networks. The framework we proposed is versatile, which is not only used to link prediction but also applicable to other directions in complex networks.

## 1. Introduction

Most real-life complex systems can be transformed into complex networks of nodes and edges [1,2,3,4,5], such as scientific collaboration network [6], protein network [7], and social network [8,9]. Link prediction has become the hot research in the complex network field and can be applied to the fields of biology, physics and other popular areas [10,11,12,13,14]. In general, link prediction is to infer the false links or the possibility of the real links between the nodes based on the known complex network structure information [10,11,15].

Recently, more and more attention are paid to the link prediction methods based on the network structure [16]. There are many prediction algorithms based on the similarity of local information, such as Common Neighbor (CN) index [10], Adamic-Adar (AA) index [17], and Resource Allocation (RA) index [18]. However, these methods only consider the structural features of the network and do not consider the weight information.

Complex networks are composed of nodes and edges [4]. If there is an edge between two nodes, it means there is a relationship between them and according to the actual situation, the edge can also be given a weight [19]. For example, the weight between two nodes in the US air transportation network indicates the frequency of flights between two airports. Compared with unweighted networks, weighted networks emphasize on the weight characteristics of the edges, so that the relationship between the nodes is not only in the form of yes or no, which further enriches the relationship between nodes, and thus weighted networks provide more research space for characterizing the complexity and non-trivial nature of complex networks [20]. On the link prediction of weighted networks, Lü et al. proposed some prediction indices based on local information, such as Weighted Common Neighbors (WCN), Weighted Adamic-Adar index (WAA), Weighted Resource Allocation (WRA) and so on [21].

Based on these methods, Yang et al. used the strength relationship between the paths of the network to perform link prediction [22]. Some researchers also predicted the weights of networks [23,24]. However, the role of topological structure and weight correlation [25] cannot be clearly identified in the link prediction based on these evaluation indices. In this study, we will propose a method to quantify the role of topology and weight correlation in link prediction using the null model knowledge. Because the weights are an important feature of weighted networks, which can indicate the strength of edges and reflect some real information, therefore, this paper also focuses on the role played by links with different weights in link prediction.

Researchers often use statistics to characterize the nontrivial nature and complexity of weighted complex networks, but most of the existing statistics only have absolute values without units, so they can not accurately characterize the network. In this paper, we use the rich club phenomenon [26] of the network and different assortativity mixing pattern [27] to construct the null model, and use the proposed framework to quantitatively analyze the influence of different statistics on link prediction.

In this study, we propose a new framework with the knowledge of null models to characterize the impact of network structure and properties on link prediction. We mainly carry out the following aspects of the study. First, we construct the corresponding null models according to the different properties of weighted networks. Then, we study the topological structure and the weight of weighted networks, and analyze their influence on link prediction. Next, we divide all ties into strong and weak ties and analyze the impact of different weights on link prediction and their role in the prediction. Finally, two important statistics, the rich club phenomenon and the assortativity mixing, in weighted networks are selected, and studied separately. In the experimental part of this paper, we apply this research framework to the empirical weighted network for analysis.

The paper is organized as follows: in Section 2, we introduce the empirical networks, basic knowledge and evaluation indicators of link prediction. In Section 3, we describe the three null model algorithms of weighted networks and analyze their relationships. In Section 4, the results of the link prediction between the original network and its null models are compared; and the effects of different strength ties on the link prediction are analyzed. In Section 5, two null networks based on rich-club phenomenon and the results of them in link prediction are described. In Section 6, we describe the assortativity and disassortativity null models and the results of them in link prediction. In Section 7, the conclusions are presented.

## 2. Data and Methods

### 2.1. The Description of the Empirical Networks

In this study, we provide a paradigm that can quantitatively examine the impact of topological structures and properties of weighted complex networks on link prediction. Here, we select seven empirical networks for experimental analysis to test this paradigm.

The *USAir* is the US air transportation network. The network consists of 332 nodes and 2126 links. The nodes represent the airports, and the links indicate the routes. The weight of a link is the frequency of flights between two airports [28].

The *Celegans* is a neural network of the worm Caenorhabditis elegans, which has 297 nodes and 2148 links, where nodes denote the neurons and links represent synaptic contacts. The number of synapses between two neurons is the link’s weight [29].

The *Lesmis* is a coappearance network of characters in the novel Les Miserables. This network has 77 nodes and 254 links. The nodes represent the characters in the novel, if two characters co-appear in the same chapter, the corresponding nodes will have an edge and their frequency is the weight of the edge [30].

The *NetScience* is a co-authorship network of scientists working on network theory and experiment. It consists of 1461 nodes and 2742 links, which denote the scientists and the relationship between their cooperation, respectively. The weight of a link represents the number of cooperation between two corresponding scientists [3,4].

The *Geom* is a collaboration network in computational geometry. The network with 7343 nodes and 11,898 links means author X wrote joint works with author Y, and the weight is the number of joint works [28].

The *CScientists* is also a co-authorship network, but it is the scientists posting preprints on the field of condensed matter in 1995–1999. It has 16,726 nodes and 47,594 links [6].

The *CatCortex* is a cat cortex network. The network consists of 95 nodes and 1170 links. The nodes represent all cortical and thalamic areas, the links stand for the connections of them [23].

The *Football* is a football players market network, which has 35 nodes and 118 links. The nodes represent the countries and the links represent players are traded in both countries. The weight is the number of players who are traded [28].

**Figure 1:**
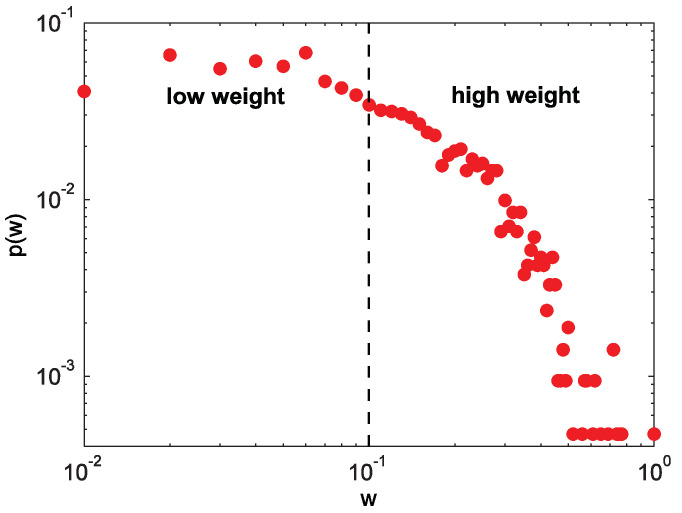
(color online) The weight distribution of the USAir network.

The USAir network is taken as an example, and the weight distribution of this network is shown in Figure 1. It can be seen that the weight distribution of this network obeys a long-tail distribution, that is to say, only a few of the edges have high weights. According to the weights of this network, a threshold is chosen to divide all edges into strong ties and weak ties, each of which accounts for 50% of the number of total edges (the dashed line in Figure 1 is the cut-off).

### 2.2. Unweighted Similarity Indices Based on Local Information

Recently, similarity indices based on local information that have the advantage of computational complexity receive more attention in link prediction, and are suitable to predict large-scale complex networks. The most commonly used similarity indices based on local information are Common Neighbor (CN) [10], Adamic-Adar (AA) [17] and Resource Allocation (RA) [18]. Therefore, we concentrate on these indexes, whose definitions are as following.

In common sense, the greater the amount of co-neighbors between the two nodes *x* and *y* are, the higher the probability of a connection between them becomes. Therefore, in the social network, it becomes that the more the number of common friends the two individuals have, the greater the probability of knowing between them is [31]. For another example, in the scientific collaboration network, the greater the number of previous cooperation between the two scientists, the greater the likelihood of the next collaboration [32]. CN indicators can be used to calculate the number of two nodes’ common neighbors [10]:

(1) $$S_{xy}^{CN}=\left|\Gamma \left(x\right)\cap \Gamma \left(y\right)\right|,$$

where Γ(x) denotes the set of neighbors of node *x*.

The AA algorithm considers that low-degree common neighbors contribute more in link prediction. For example, in Facebook, the individuals who have more followers are often prominent figures in a certain area. Then, there may not be similar interests among individuals who share their concerns. Conversely, if two individuals are concerned about an individual with a small number of followers, there is a great deal of similarity between them or they may be in a social circle. The AA indicator is to further consider the degree information between two nodes, to give a weight for each node according to the degree of common neighbor nodes. The formula is as follows [17]:

(2) $$S_{xy}^{AA}=\sum_{z\in \Gamma (x)\cap \Gamma (y)}\frac{1}{\log k(z)},$$

where k(z) is the degree of node *z*.

RA index is a link prediction index based on node local structure information proposed in [18], from the perspective of resource allocation in complex networks. Considering the two nodes *x* and *y* are not connected directly, the node *x* wants to pass information to the node *y* only through their common neighbors. Common neighbors also become their resource transmitters. The number of resources received by node *y* is the value of the RA indicator, as shown below:

(3) $$S_{xy}^{RA}=\sum_{z\in \Gamma (x)\cap \Gamma (y)}\frac{1}{k(z)}.$$

The significant difference between the RA indicator and the AA indicator is the way in which weights of common neighbor nodes are given, that is, the former decreases in the form of $\frac{1}{k}$, while the latter decreases in the form of $\frac{1}{\log k}$. It can be seen that in a network with a small average degree, there is little difference between RA and AA. Nonetheless, if a network possesses a large average degree, there are significant differences between RA and AA [33]. The study reveals that the RA indicator is better than the AA indicator in the applications that characterize the performance of weighted networks [34] and community mining [35]. Soundarajan and Hopdroft proposed in [36] that meso-scale structures such as community can be used to improve local indicators, among which the RA indicator is most effective.

### 2.3. Weighted Similarity Indices Based on Local Information

The CN, AA and RA indicators which apply to an unweighted network can be extended to the network with weights. They are called WCN, WAA and WRA [21], as shown in Equations (4)–(6):

(4) $$S_{xy}^{WCN}=\sum_{z\in \Gamma (x)\cap \Gamma (y)}{W(x,z)}^{\alpha }+{W(z,y)}^{\alpha },$$

(5) $$S_{xy}^{WAA}=\sum_{z\in \Gamma (x)\cap \Gamma (y)}\frac{{W(x,z)}^{\alpha }+{W(z,y)}^{\alpha }}{\log(1+s(z))},$$

(6) $$S_{xy}^{WRA}=\sum_{z\in \Gamma (x)\cap \Gamma (y)}\frac{{W(x,z)}^{\alpha }+{W(z,y)}^{\alpha }}{s(z)},$$

where w(x,y) is the weight of the edge between node *x* and node *y*. Compared with the three unweighted network indexes defined above, the revised three indexes mainly introduced a new free parameter α, which is used to adjust the role of weight in link prediction. When α=0, weighted indexes will return to unweighted form. When α=1, weights in these indexes will be the real weights of networks. When α>0, weights of networks will be magnified and when α<0, it will be reduced. In the experimental part, we chose the different values of α in the above cases, respectively, in order to observe the performance of different strength weights in link prediction. Because the results of WCN, WAA and WRA are similar in our experimental networks, we only show the results of WCN algorithm in the following sections.

### 2.4. Two Evaluation Indexes of Link Prediction

Considering a simple undirected network G(V,E), *V* and *E* represent the set of nodes and edges in the network, respectively. First, we select two nodes x,y∈V, and then calculate a score $S_{xy}$ according to the definition of indicators. For the edges, we divide the existing edges into test sets $E^{T}$ and training sets $E^{P}$, accounting for 10% and 90% of the total number of edges each. Then, we take the edges that do not exist in the network as the prediction set, $E^{N}$, where the number of edges is the same as the test set. Clearly, $E^{T}\cup E^{P}=E$, $E^{T}\cap E^{P}=\emptyset$, and $E^{N}\notin E$. Next, we introduce two indicators to evaluate the accuracy of link prediction from two different perspectives.

The Area Under the receiver operating characteristic Curve (AUC) focuses on the overall accuracy of the measurement [37]. It can be interpreted as the probability that scores of edges of test set are higher than scores of randomly non-existent edges. The specific calculation method is to take a random score from the scores of the test set and those of the non-existent edges, respectively. Then, compare two scores. If the score of the test set is higher, add 1 point; if two scores are equal, add 0.5 points. Finally, calculating the average score [21], the AUC index is defined as:

(7) $$AUC=\frac{n^{\prime }+0.5n^{\prime \prime }}{n},$$

where $n^{\prime }$ denotes the number that the score of the test set is higher, and $n^{\prime \prime }$ is the number that two scores are equal, and *n* is the total number of all scores.

Precision evaluation index is the scores of the edges are sorted in descending order. If the first *L* of the scores contain the number of scores of the test edges being *m*, the Precision is defined as [21]:

(8) $$Precision=\frac{m}{L},$$

where *m* means the times that *L* maximum edge values appear in the test section, and *L* represents the number of the selected maximum edge values. The larger the *m* is, the more accurate the prediction is. The Precision is more focused on the scores of the top *L* values [26].

## 3. The Null Models of Weighted Networks

As with unweighted networks, a weighted network can also be fitted and characterized by using some null models. However, the existing configuration models are not involved the study of weight distribution. Therefore, it is hard to use them as null models of a weighted network. Although there have been several classical models in the study of weighted networks, such as the weighted network model proposed by Barrat et al. [38] and the traffic flow driven model proposed by Wang et al. [39], it is not convenient to analyze the correlation between topological structure and weight distribution of weighted networks by using these models. It is very difficult to analyze the impact of these two factors on the nature of a network.

The weight-topology correlation [25] refers to the existence of a certain correlation between the topology of a network at different levels and the weight distribution. This correlation may exist in local structure or global topology structure. This feature often plays a major role in optimizing allocation of resources in complex systems [40]. A variety of null models based on the original network can precisely control several critical factors, which affect the weight-topology correlation. Therefore, we discuss three null models for the weight-topology correlation below.

### 3.1. The 1k Null Model

**Figure 2:**
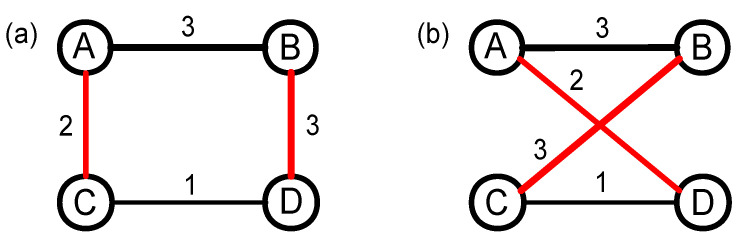
(color online) The constructing process of the 1k null network based on random swapping edges. (**a**) the topology structure of the original network; and (**b**) the topology structure of the 1k null network after random swapping two edges.

The 0k null model changes not only the topology structure, but also the degree distribution of the original network, which leads to the strong randomness. Actually, the strong randomness makes the 0k null model used less in studying real-life networks. Because the 1k null model maintains the degree distribution of the original network, it has been used in rich-club [41] and community [42] detections. The 1k null model of the weighted network can also be generated using the way of random disconnecting and rewiring, and the specific constructing process has been shown in Figure 2. Firstly, nodes A and B, and nodes C and D are connected as in Figure 2a, while nodes A and D, and nodes B and C are not connected. Then, we disconnect the links AB and CD, and rewire nodes A and D, and nodes B and C, respectively. Finally, the weight of the newly added links AD (BC) is the same as the weight of links AC (BD) in the original network. Generally, it should be emphasized that we need to repeat the above process many times until the original network is fully randomized. Obviously, the repeating time is correlated with the size of the network (i.e., the number of edges *N*), so the repeating time is set to be 2∗N in this study.

### 3.2. The Structure-Shuffling Null Network

**Figure 3:**
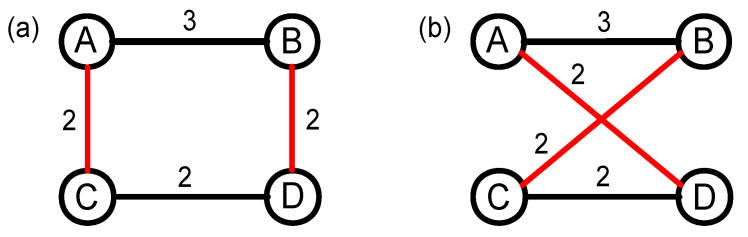
(color online) The constructing process of the structure-shuffling null network based on random swapping edges. (**a**) the topology structure of the original network; and (**b**) the topology structure of the structure-shuffling null network after random swapping two edges.

The 1k null network destroys both structure correlation and weight correlation of the original network, so, based on this model, the impact of each of the two correlations (structure or weight) on link prediction cannot be studied separately. Therefore, we introduce the structure-shuffling network to only destroy the topology structure of the original network, but to preserve its weight distribution. The specific constructing process has been shown in Figure 3. Firstly, nodes A and B, and nodes C and D are connected in Figure 3a, while nodes A and D, and nodes B and C are not connected. Then, we randomly select of a pair of the links AC and BD that have equal weights. Finally, we disconnect the links AC and BD, and rewire nodes A and D, and nodes B and C, respectively. After the whole operation, the weight distribution of each node in the network is kept, even though the topology structure of the network has been changed. In some networks, it is difficult to find enough links that have the same weights to shuffle. In order to ensure that as many as possible edges can be swapped, an alternative method is adopted to exchange the edges with approximate weights.

### 3.3. The Weight-Shuffling Null Network

**Figure 4:**
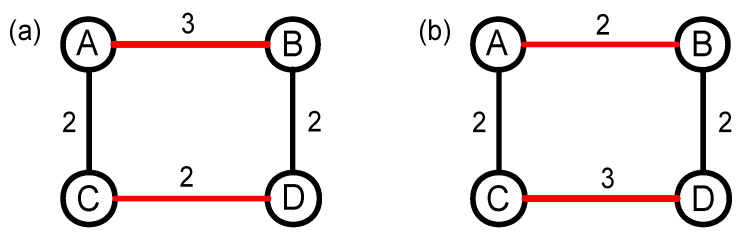
(color online) The constructing process of the weight shuffling null network based on random swapping edges. (**a**) the topology structure of the original network; and (**b**) the topology structure of the weight-shuffling null network after random swapping two edges.

In addition to topology structure correlation, weight correlation between edges of a node is the other important factor that affects the nontrivial nature of complex networks. In [43], the authors proposed a weight-shuffling null model to study the impact of weight correlations. The constructing process is shown in Figure 4. First of all, we randomly select two edges that have different weights, such as edges AB and CD. Then, the weights of these two links are shuffled, so now the weight of AB is 2, and the weight of CD is 3. Here, we do not change the topology structure of the original network, but change the weight correlation of the four nodes. According to the size of the network, we need to repeat the operation above many times to let the weight correlations of the null network randomized enough.

### 3.4. The Relationship between Three Null Models

**Figure 5:**
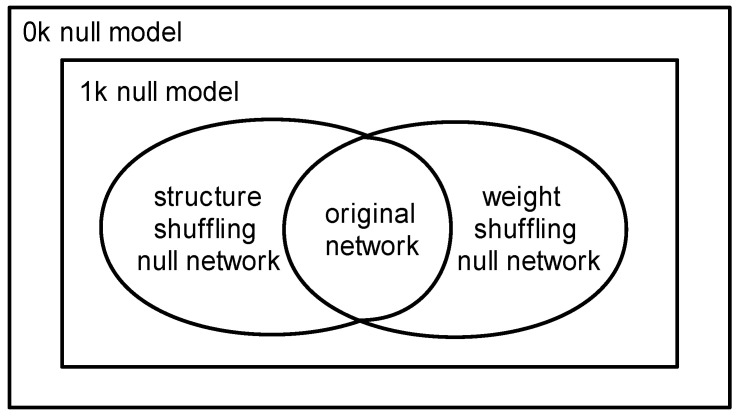
The relation between null models of a weighted network.

Considering the existence of topological structure and weight distribution in the original undirected weighted network, we describe the relationship between several null models of the weighted networks, as shown in Figure 5. The 0k and 1k null models randomize original network with two levels of weight and topology, which have the highest randomness. The 0k null model only guarantees average strength of a weighted network unchanged, while the 1k null model also needs to guarantee the degree distribution of nodes is constant. Therefore, the 0k null model has the strongest randomness. Because of the strong randomness of a null model which can destruct more factors, we select the 1k null model whose randomness is weaker than the 0k null model. The simplest scrambling algorithm on a weighted network is to construct the 1k null model. Because the topology and the weights of the edges are both randomized at the same time, it is not clear whether the network’s essential nature is due to the topology structure or the weight distribution. Hence, we have to introduce the structure-shuffling null network and the weight-shuffling null network.

The structure-shuffling null network is to randomize the structure of the network, keeping the degree and weight distribution of the network unchanged. The weight-shuffling null network is to randomize weight distribution, ensuring that the topology structure of a network remains unchanged. Because both of these null models have only randomly one aspect of the network (topology structure or weight distribution), they have a lower randomness. By comparing the dissimilarity between the above two null networks and the original network, we can draw a clear conclusion of the role of the topology and weight.

**Figure 6:**
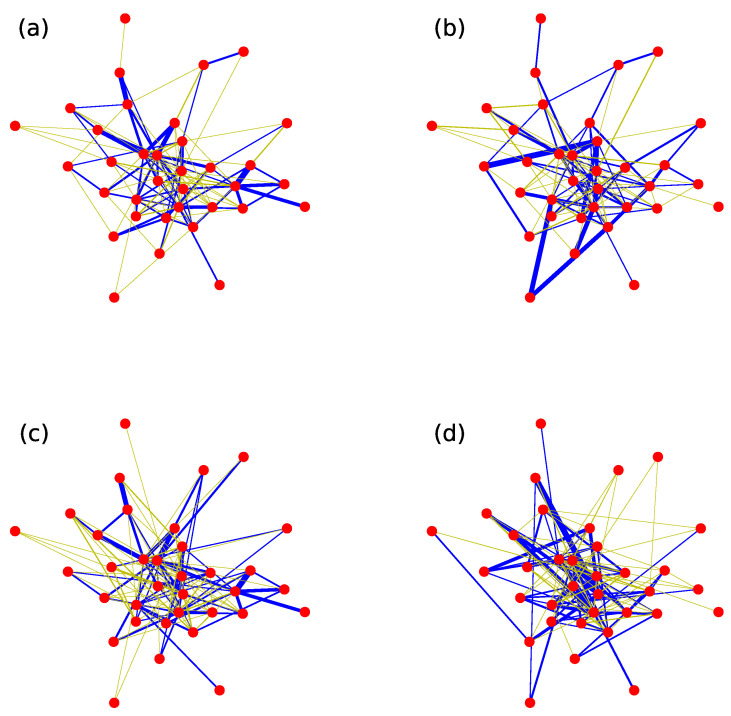
(color online) The visualization of the football network and its three null models. (**a**) the original network; (**b**) the weight-shuffling null network; (**c**) the structure-shuffling null network; and (**d**) the 1k null network.

In order to demonstrate the differences of an original weighted network and its three null models more specifically, we have chosen an empirical network, football network, to visualize, as shown in Figure 6. The weight-shuffling null model is constructed by random scrambling weights in Figure 6b. The structure-shuffling null model is constructed by random scrambling two links with equal weights repeatedly in Figure 6c. The 1k null model is constructed by random scrambling two links repeatedly in Figure 6d. From the figure, we can clearly observe that the weight-shuffling null model destroys the weight correlation and the topology of the original network is preserved. The structure-shuffling null model is exactly the opposite process. This figure also shows that 1k null model has the strongest randomness.

## 4. Link Prediction Based on Null Models of Weighted Networks

### 4.1. The Impact of Topology Structure and Weight on Link Prediction

**Figure 7:**
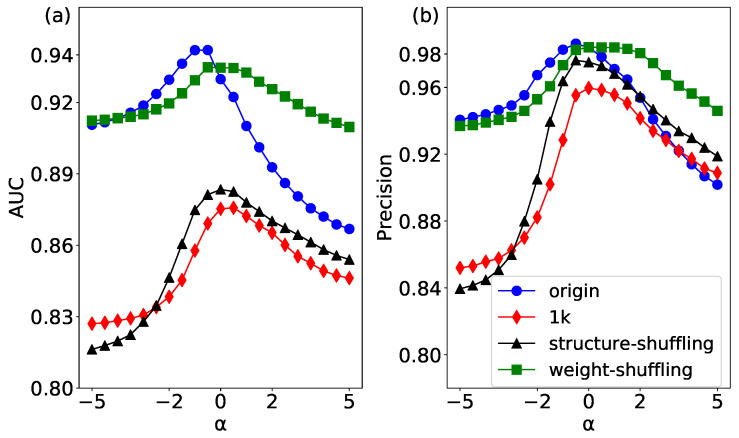
(color online) The link prediction results of the USAir network and its three null networks by using the WCN algorithm. (**a**) the results of AUC; and (**b**) the results of Precision.

The link prediction results of the original network and its corresponding three null models have been shown in Figure 7. The original network has the best accuracy when the parameter α is less than 0, which indicates that weak links may play an important role in the link prediction [44]. In this study, we consider two kinds of edge correlation: weight correlation and degree correlation. The 1k null networks only keep the degree distribution of the original network, and destroy both weight correlation and degree correlation. Therefore, the AUC and Precision of 1k null networks show the lowest prediction accuracy, which validates that it has the strongest randomness of the original network.

In the structure-shuffling network, two edges with the same weight are disconnected and rewired. The above operations can destroy the topology structure of the whole network. In particular, it can completely change the degree correlation of the edges. The AUC and Precision of the structure-shuffling network is only higher than that of 1K model, which means the degree correlation has a relatively strong effect on link prediction.

In the weight-shuffling network, we only swap the weights of two edges without changing the weight correlation so as not to change the link relationship of the whole network. It can be seen that the results of link prediction in the weight-shuffling network are close to the original network, which implies that the weight correlation has a low effect on link prediction. Interestingly, when the parameter α is more than 0, the AUC and Precision of the weight-shuffling network are higher than those of the original network. The reason is that there are many weak ties that play a strong role in the original network, and by shuffling the weights of these edges, the prediction effect is better than the original network when the weight increases. In summary, our results show that both weight correlation and degree correlation have an impact on link prediction of a network, and the degree correlation has a stronger effect than the weight correlation.

Several other empirical networks whose results of link prediction based on three null models are given in the form of maximums of AUC and Precision, as shown in Table 1. In these networks, the three null models are provided that can quantify topology structure and weight correlation in link prediction of weighted networks. All results of 1k null model are the worst, and the prediction results of the weight-shuffling null model are better than that of the structure-shuffling null model. To the *Celegans*, the AUC and Precision of weight-shuffling null model are better than the original networks. In the *Gemo* and the *CScientists*, the Precision of weight-shuffling null model are equal to the original networks. In the rest of results, the original networks have the best performance compared with their corresponding three null models.

### 4.2. Predicting Strong and Weak Ties

**Figure 8:**
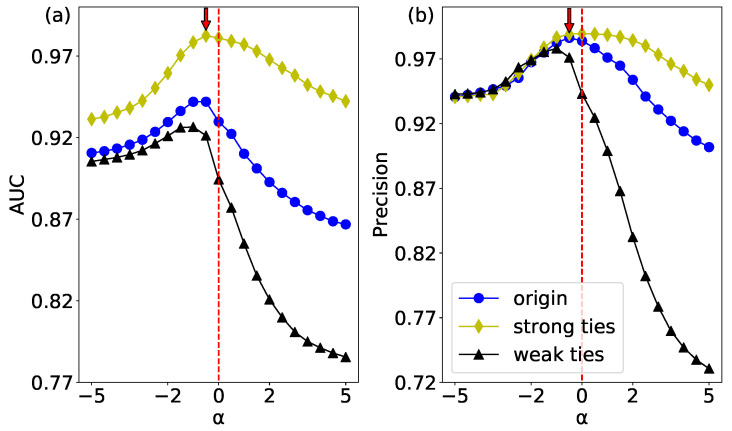
(color online) The WCN prediction results of the USAir network’s links with different weights. (**a**) the results of AUC; and (**b**) the results of Precision.

The significant difference between the weighted network and unweighted network is that the edges in weighted network have a meaningful weight. The role of the weight in link prediction is a question worth considering, especially whether there is a strong effect of weak ties in weight link prediction. Aiming at this problem, we sort all the edge weights in a descending order, and the first 50% are considered to be strong ties and the other 50% as weak ties in Figure 1. In addition, we randomly selected half of edges for link prediction. According to the results of AUC and Precision in Figure 8, the highest performance can be obtained at α=−0.5<0, which means that there is a “strong effect of weak ties” in the *USAir* network. Because when α is less than 0, the weights of the weak ties become larger, and the result of the original network is the most accurate, which shows that the weak ties play a strong role in link prediction. Moreover, strong links perform the highest accuracy, and weak links get the lowest performance. This result indicates that the edges with heavy weights (strong ties) are easier to be predicted than the edges with small weights (weak ties).

The results of link prediction of strong and weak ties for other empirical networks are given in the form of maximums of AUC and Precision, as shown in Table 2. The results show that in the *USAir*, the *Celegans*, the *Gemo*, the *Lesmis* and the *CatCortex*, the strong ties are easier to predict, but there is a few networks whose weak edges are predicted more easily, such as the *Netscience* and the *CScientists*.

### 4.3. The Strong Effect of Weak Ties

**Figure 9:**
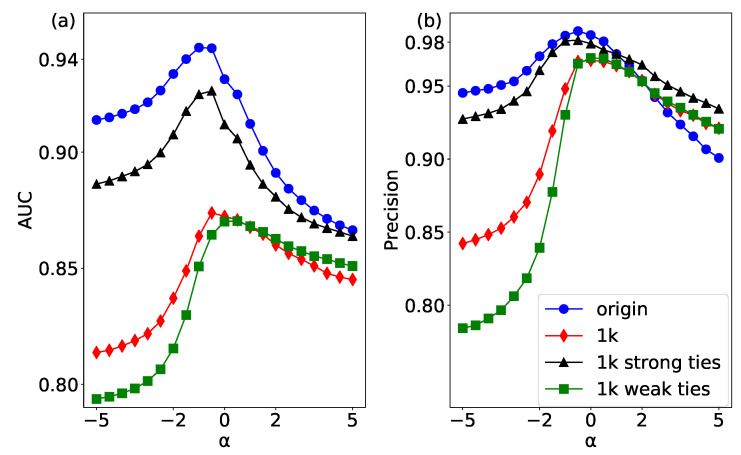
(color online) The WCN prediction results of the USAir network and its null networks with 1k scrambling of the different weights. (**a**) the results of AUC; and (**b**) the results of Precision.

In order to study the role of different weights in link prediction, we construct the 1k null model of strong links, weak links and random selecting links, and then predict the network. The prediction results of the original network and its 1k null model of the different weights are shown in Figure 9. From the result of AUC, it can be seen the accuracy of the original network is higher than that of the networks after 1k random swapping, which indicates random scrambling of the network increases its randomness and reduces the accuracy of the prediction results. By observing the results of different weights after scrambling, it can be found that the prediction accuracy of the weak ties scrambling is the lowest. It is further explained that the weak ties play a greater role in the prediction of the original network than the others.

The results of link prediction of the original network and the null networks with 1k scrambling of the different weights for several other empirical networks are given in the form of maximums of AUC and Precision, as shown in Table 3. The bold parts in Table 3 are the edges with poorer performance after 1k scrambling, which means that these edges play an important role in the link prediction. The *USAir*, the *Netscience* and the *CScientists* have the strong effect of weak ties; however, the rest of the networks have the strong effect of strong ties.

## 5. Link Prediction in the Null Network With Rich-Club Phenomenon

### 5.1. The Null Networks Based on Rich-Club Phenomenon

Many complex networks have a power-law form of degree distribution, so they are called scale-free networks. In these kinds of networks, a few nodes that have far more links than others are called rich nodes or hub nodes. If rich nodes tend to connect each other, it is referred to the rich-club phenomenon. In weighted networks, we define nodes that have strong strength as rich nodes. The connections between them are called the weight rich-club phenomenon [26].

**Figure 10:**
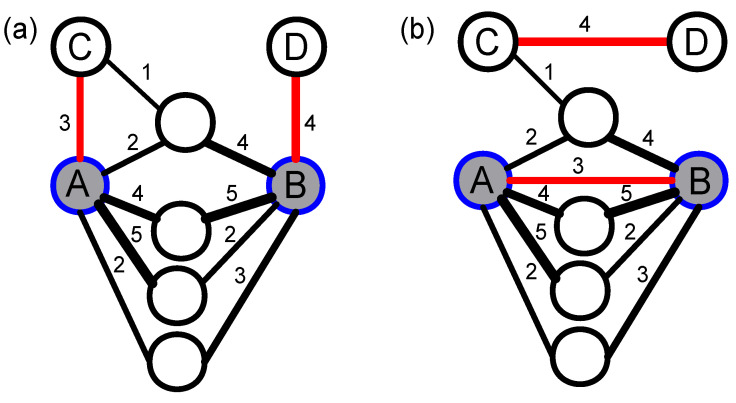
(color online) The constructing process of a null net-work with a rich-club phenomenon based on disconnecting- rewiring. (**a**) an original network without a rich-club phenomenon (rich nodes A and B are not connected); and (**b**) the topology structure of the null network with a rich-club phenomenon after disconnecting and rewiring.

As shown in Figure 10a, because there is no connection between two rich nodes A and B, the network does not have a rich-club phenomenon. To generate a null network with a rich-club phenomenon, we need to disconnect and rewire specific edges as follows. First, we randomly select one edge of node A and B respectively, ensuring that the two edges are connected to non-rich nodes such as C and D (nodes C and D should not be connected). Then, we disconnect the edges AC and BD, and connect rich nodes A and B, and unrich nodes C and D, respectively. Now, rich node A is connected to another rich node B, so the network has a rich-club property (as shown in Figure 10b). The algorithm described how to make the networks have rich-club characteristics. If the above process is used inversely, the null network almost without rich-club characteristics can be constructed.

### 5.2. The Impact of the Rich-Club Phenomenon on Link Prediction

**Figure 11:**
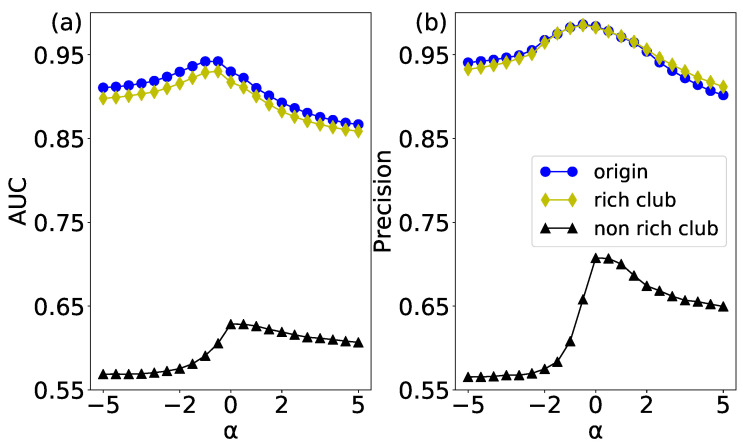
(color online) The WCN prediction results of the USAir network and its rich-club and non-rich-club null networks. (**a**) the results of AUC; and (**b**) the results of Precision.

The link prediction results of the original network and its corresponding two null models have been shown in Figure 11. According to the AUC results in Figure 11a, the sorted performances from high to low are the original network, rich-club null model, and non-rich-club null model. Conjecturing the reason is as follows. The original network has a non-trivial topology structure, so we can get a relatively high predicting performance. When null models are constructed (either rich-club or non-rich-club), the disconnecting-rewiring operation of edges will destroy the non-trivial network structure in the original network. Therefore, the AUC results of rich-club and non-rich-club null models are lower than that of the original network. Especially, the non-rich-club null model shows the lowest performance, for it introduces more random elements into the original network.

The precision evaluation index is mainly used to measure the connection of high scores in a network. The precision results of the original network and its corresponding null networks are shown in Figure 11b. Interestingly, the results of the rich-club null model are basically the same as the original network. It validates the existence of the rich-club phenomenon in the original network. However, the non-rich-club null network breaks the connection between rich nodes, whose randomness is higher than the rich-club null network and the original network. Therefore, the precision of the non-rich-club null network is the lowest.

## 6. Link Prediction in the Null Network with Assortativity and Disassortativity

### 6.1. The Assortativity and Disassortativity Null Networks

In unweighted networks, degree correlation is an important feature. The actual complex networks have the correlation between degrees rather than completely irrelevant. Such as in the study of protein-mediated network and gene regulatory network, Maslov et al. found that the connection between the big-degree nodes is suppressed by the system, and the possibility of connection between high-degree nodes and low-degree nodes is greater [45]. Extending to weighted networks, we can study the correlation between node strength instead of the degree. If the assortativity mixing pattern between the strength of the original network is wanted to be changed, it is necessary to disconnect and reconnect tendentiously to construct the corresponding assortativity or disassortativity [27] null networks.

**Figure 12:**
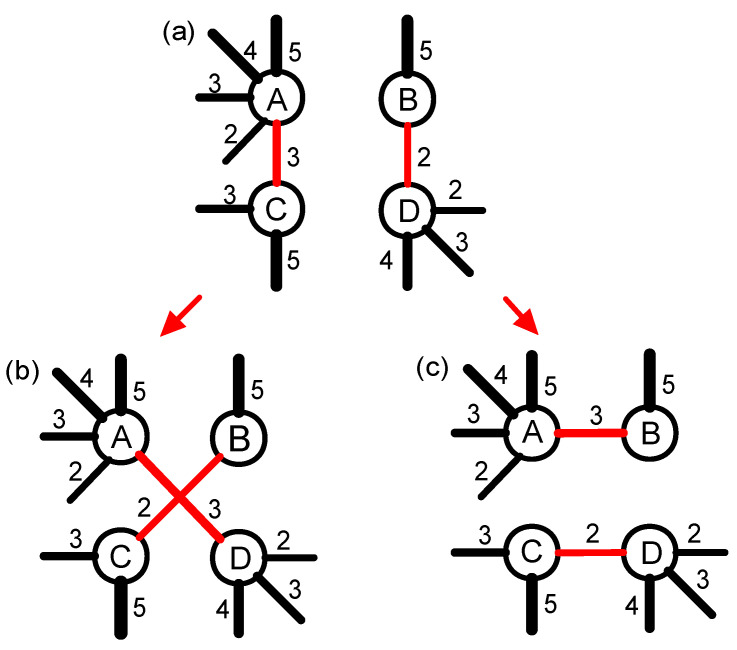
(color online) The constructing process of assortativity and disassortativity null networks based on disconnecting-rewiring edges. (**a**) the topology structure of the original network; and (**b**) the topology structure of the assortativity null network after disconnecting and rewiring edges; and (**c**) the topology structure of the disassortativity null network after disconnecting and rewiring edges.

As shown in Figure 12a, and the original network has four nodes which have different strengths, the order of strength is A, D, C and B. If we connect the two nodes with the strongest strengths (A and D), and the two nodes with weakest strengths (B and C), we can get an assortative network. This process can be repeated until we generate the strongly positive assortativity null network. On the contrary, if two pairs nodes of different strengths are connected respectively (A and B, C and D), it will enhance the disassortativity of the original network. This process can be repeated until we generate a strongly disassortative null network.

### 6.2. The Impact of Assortativity Mixing Patterns on Link Prediction

**Figure 13:**
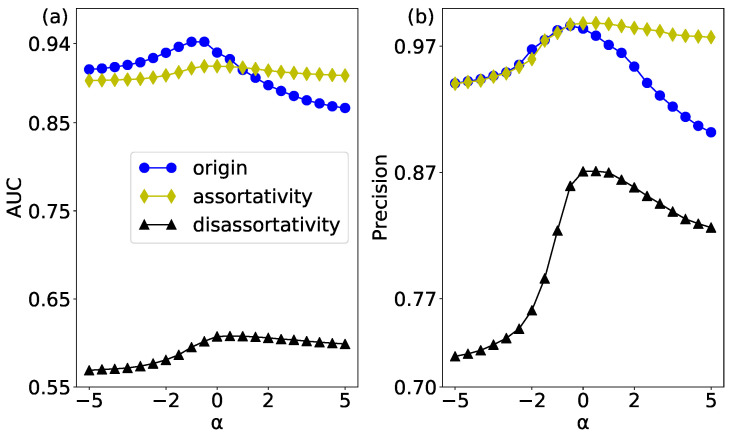
(color online) The WCN prediction results of the USAir network and its two null networks. (**a**) the results of AUC; and (**b**) the results of Precision.

The link prediction results of the original network and its two null networks whose assortativity mixing pattern is changed have been shown in Figure 13. In the process of construction, the assortativity and disassortativity null networks have destroyed the correlation of weights and degrees in the network, which has enhanced the randomness relative to the original network.

In Figure 13a, we show the prediction result of the AUC evaluation index. It can be seen that the assortativity null network enhances the assortativity mixing pattern of node strength in network, so its prediction results are relatively stable, which is not affected by the weight. The disassortativity null network has the highest randomness, so the prediction accuracy is the lowest. The prediction result of Precision evaluation is shown in Figure 13b. In the process of constructing the assortativity null network, the high-strength nodes are connected to each other, so these edges are higher than the other edges in the original network. Therefore, when measuring the accuracy with Precision evaluation index, some results of the assortativity null networks are higher than the original network.

## 7. Conclusions

In this study, we have mainly studied how to measure various factors in link prediction of weighted networks. We provide a general framework that quantifies different factors by constructing a set of null models. We apply the framework of null models to link prediction of weighted networks and do the following works. It also can be used in the study of describing the randomness of weighted network, social networks communication, and so on.

One of the most important properties of weighted networks is the topology-weight correlation. In order to test this property and distinguish the role between topology and weight in link prediction, we have constructed three types of null models in weighted complex networks. It can be used to detect the topology-weight correlation in weighted complex networks, and this framework can be used to quantitatively detect the roles of topology and weights in link prediction. Through the experiment of the empirical networks, it can be seen that there is a topology-weight correlation in these networks, and the topology plays a greater role in the prediction than the weights. We study weights, which is an important feature of weighted networks and the impact of different weighted ties on link prediction by dividing all ties into strong and weak ties. It is verified that there is a strong effect of weak ties in weighted networks, such as the *USAir*, the *NetScience* and the *CScientists*. For the link prediction of the links with different weights, the strong ties in most networks are easier to predict. However, the weak edges can be predicted more easily for a few networks, such as the *Netscience* and the *CScientists*.

In the work of characterizing nature of weighted networks, by constructing different forms of null models and combining statistics, we can more accurately characterize complexity and non-trivial nature of networks. In this study, null models about the rich-club phenomenon and the different strength assortavity characteristics have been constructed. Through the experiment on the empirical network, it is found that there is the rich-club phenomenon in the original network, and the non-rich club network has the strongest randomness. When the network has a strong assortativity of node strength, the network predict results are relatively high and not affected by the change of weights. In this study, this general framework is proposed to describe the characteristics of the network more accurately. Our findings in this work can be used to improve the performance of other applications in weighted complex networks.

## Figures and Tables

**Table 1:** The maximums of the link prediction results of the original network and their three null networks by using the WCN algorithm. The *USAir* is the US air transportation network [28], the *Celegans* is a neural network of the worm Caenorhabditis elegans [29], the *Lesmis* is a coappearance network of characters in the novel Les Miserables [30], the *NetScience* is a co-authorship network of scientists working on network theory and experiment [3,4], the *Geom* is a collaboration network in computational geometry [28], the *CScientists* also a co-authorship network [6], and the *CatCortex* is a cat cortex network [23].

		*USAir*	*Celegans*	*Netscience*	*Gemo*	*Lesmis*	*CatCortex*	*CScientists*
AUC	Origin	0.942	0.831	0.937	0.889	0.878	0.781	0.952
	Weight-shuffling	0.935	0.832	0.935	0.888	0.865	0.763	0.951
	Structure-shuffling	0.883	0.679	0.634	0.573	0.703	0.697	0.586
	1k	0.876	0.668	0.51	0.529	0.710	0.688	0.504
Precision	Origin	0.986	0.94	0.997	0.999	0.911	0.925	0.999
	Weight-shuffling	0.984	0.967	0.993	0.999	0.917	0.911	0.999
	Structure-shuffling	0.976	0.903	0.808	0.676	0.826	0.919	0.692
	1k	0.960	0.915	0.525	0.579	0.807	0.903	0.510

**Table 2:** The maximums of the WCN prediction results of the links with different weights.

		*USAir*	*Celegans*	*Netscience*	*Gemo*	*Lesmis*	*CatCortex*	*CScientists*
AUC	Strong ties	**0.983**	**0.891**	0.912	**0.925**	**0.943**	**0.810**	0.935
	Weak ties	0.927	0.799	**0.999**	0.882	0.789	0.780	**0.999**
Precision	Strong ties	**0.989**	**0.955**	0.996	**0.999**	**0.913**	**0.921**	0.999
	Weak ties	0.978	0.916	**0.998**	0.999	0.880	0.909	**0.999**

**Table 3:** The maximums of the WCN prediction results of the empirical networks’ null networks with 1k scrambling of the different weights.

		*USAir*	*Celegans*	*Netscience*	*Gemo*	*Lesmis*	*CatCortex*	*CScientists*
AUC	1k Strong ties	0.916	**0.689**	0.726	**0.700**	**0.748**	**0.696**	0.756
	1k Weak ties	**0.862**	0.715	**0.702**	0.751	0.838	0.746	**0.732**
Precision	1k Strong ties	0.956	**0.906**	0.987	**0.907**	**0.852**	**0.907**	0.999
	1k Weak ties	**0.941**	0.909	**0.952**	0.923	0.895	0.919	**0.997**
